# Interleukin-1 Two-Locus Haplotype Is Strongly Associated with Severe Chronic Periodontitis among Yemenis

**DOI:** 10.1155/2012/231309

**Published:** 2012-06-19

**Authors:** Nezar Noor Al-hebshi, Amat-alrahman Ahmed Shamsan, Mohammed Sultan Al-ak'hali

**Affiliations:** ^1^Molecular Research Laboratory, Faculty of Medical Sciences, University of Science and Technology, Sana'a, Yemen; ^2^Faculty of Dentistry, Jazan University, P.O. Box. 114, Jazan, Saudi Arabia; ^3^Department of Periodontology, Faculty of Dentistry, University of Sana'a, Sana'a, Yemen

## Abstract

*Aim*. To assess IL-1A C[−889]T and IL-1B C[3954]T genotypes as well as haplotypes in relation to sever chronic periodontitis (SCP) among Yemenis. *Materials and Methods*. 40 cases with SCP and 40 sex- and age-matched controls were included; all were nonsmokers and free of systemic diseases. Genotyping at each locus was performed using an established PCR-RFLP assay. The Haploview and SimHap software were used to assess data for Hardy-Weinberg's equilibrium (HWE) and linkage disequilibrium (LD) and to obtain subject-level haplotypes. Multiple logistic regression was used to seek for associations in dominant, additive, and recessive models. *Results*. Mean plaque index (MPI) showed the strongest association with SCP (OR = 16). A significant LD was observed in the cases (*D*' = 0.80 and *r* ^2^ = 0.47). The genotype at each locus showed significant association with SCP in the recessive model (TT versus TC + CC) even after adjustment for MPI (OR = 6.29 & 461, resp.). The C-T haplotype conferred protection against SCP in a dominant manner (OR = 0.16). On the other hand, the T-T haplotype in double dose (recessive model) showed strong association with CP (OR = 15.6). *Conclusions*. IL-1 two-locus haplotype is associated with SCP in Yemenis. Haplotype-based analysis may be more suited for use in genetic association studies of periodontitis.

## 1. Introduction

Chronic periodontitis is currently viewed as an immunologically mediated destruction of tooth supporting tissues, the periodontium, provoked by specific pathogenic bacterial consortia in subgingival biofilm [1]. However, the occurrence, extent, and severity of the destructive process are dependent on the individual's susceptibility to the disease, which is in turn influenced by risk factors independent of the microbial challenge [2]. Environmental factors, such as cigarette smoking, and systemic diseases, such as diabetes, are well-established, classical examples of such risk modifiers [3].

Lately, the role of genetics in defining host susceptibility to chronic periodontitis has drawn much attention. Initial evidence for a significant genetic element in the pathogenesis of periodontitis came from twin- and family studies, in which chronic periodontitis was shown to have around 50% heritability [4, 5]. Since then, many genes, such as the cytokine gene family, pattern-recognition receptor genes and the vitamin D receptor gene, have been explored for allelic variants that may be associated with chronic as well as aggressive periodontitis [6]. Because some cytokines, particularly the IL-1 and TNF-A proteins, are strongly implicated in the pathogenesis of chronic periodontitis [7], the genes encoding them have been more extensively investigated with the biological premise that certain polymorphisms result in hyper secretion of these proinflammatory molecules and thus in severe periodontal destruction [8].

Associations between polymorphisms of the IL-1, IL-4, IL-6, IL-10, and TNF-A genes and chronic periodontitis have been reported by several investigators; however, an equivalent number of studies failed to demonstrate such an association, making the overall evidence fragile [6]. However, the situation is somewhat more encouraging in the case of the IL-1 gene. In a recent meta-analysis of 53 publications, the IL-1A C[−889]T and IL-1B C[3954]T polymorphisms, and the so-called composite genotype [9] maintained significant associations with chronic periodontitis especially in its severe form, indicating that these polymorphisms should continue to be considered as potential “genuine” genetic determinants of chronic periodontitis [10]. However, it must be pointed out that assessment of association between the IL-1 gene cluster and chronic periodontitis has largely been based on genotype analysis only (individual SNPs or composite genotype); linkage disequilibrium or haplotype-based analyses have been nearly lacking. It is therefore recommended that these methods of analysis should be considered in future studies [10].

In the analysis referred to above, differences in effect and size between Caucasians and Asians were also noted, substantiating the view that genetic influence on chronic periodontitis may vary among ethnic groups. So far, studies on the association between the IL-1 gene polymorphisms and chronic periodontitis have mostly involved white Caucasian, Asian, and to a lesser extent, Hispanic study populations [6]. Information on such an association in other ethnic groups is sparse. The purpose of this study was, therefore, to assess the association between the IL-1A C[−889]T and IL-1B C[3954]T polymorphisms and sever chronic periodontitis among Yemenis based on haplotype analysis.

## 2. Material and Methods

### 2.1. Study Subjects

The Quanto software (University of Southern California, USA) was used to calculate sample size, assuming a population risk of 10%, an allele T frequency of 20%, a log additive mode of inheritance, and an OR of 3 for the heterozygous genotype, with a 95% level of confidence and 80% power.

Accordingly, forty subjects, ≥35 years old, with advanced chronic periodontitis (having at least one tooth site with pocket depth ≥4 mm and clinical attachment loss ≥6 in each quadrant) and forty age- and sex-matched controls (having no site with clinical attachment loss ≥3) were recruited from among patients attending the dental clinics at the Faculty of Dentistry, Sana'a University, and also a number of private dental centers in Sana'a City. All subjects were required to have Yemeni parents. Smokers and those with systemic diseases such as diabetes mellitus were strictly excluded. All teeth, except third molars, were assessed for plaque index [11], pocket depth (PD), and clinical attachment loss (CAL) at four sites per tooth by a single examiner (AAS). To be involved in the study, every subject was required to sign a written consent. The clinical features of both study groups are shown in Table 1.

### 2.2. Sampling and DNA Extraction

Oral epithelium was used as the source of genomic DNA. Each subject was instructed to rinse his/her mouth with water to remove any debris, rubber his/her cheeks against the molars and then swish with 10 mL saline for 30 seconds to obtain epithelial cells. The samples, collected in sterile containers, were then stored at −20°C. In preparation for DNA extraction, epithelial cells were pelleted by centrifuging the samples at 3000 rpm for 3 minutes, and then resuspended in 400 mL phosphate buffer saline. DNA was extracted from 200 *μ*L of the suspension using the Purelink Genomic DNA extraction kit (Invitrogen, USA) according to manufactures' instructions. Presence of valid DNA was confirmed using a human ATCB gene Taqman real-time PCR assay (Primerdesign, UK).

### 2.3. IL-1A C[−889]T and IL-1B C[3954]T Genotyping

The regions of interest (99 and 194 bp for the IL-1Aand B genes, resp.) were amplified using sequence-specific primers (Table 2). The reaction mix for each locus consisted of 18 *μ*L Platinum Blue PCR SuperMix (Invitrogen, USA), 1 *μ*L primer mix (600 nM each), and 1 *μ*L DNA sample. MgCl_2_ concentration was adjusted to 2.5 mM for the IL-1A reaction. The following thermal cycling profile was used: an initial denaturation/enzyme activation cycle at 95°C for 10 min, 35 cycles (38 for IL-1A) of denaturation at 95°C for 15 s, annealing at 55°C (54°C for IL-1A) for 30 s, and extension at 72°C for 30 s, followed by final extension cycle at 72°C for 5 min. The PCR product of the IL-1A gene was digested using 2.5 units of Nco I enzyme (Invitrogen, USA) for 3 h at 37°C, while that of the IL-1B gene was digested with 4 units of Taq I enzyme (Invitrogen, USA) at 65°C for 3 h. The restriction fragments were resolved by electrophoresis in 5% agarose gel at 50 V for 75 minutes. The genotype at each locus was determined (homozygous allele C, heterozygous, or homozygous allele T) based on fragment patterns as previously described [9].

### 2.4. Linkage Disequilibrium and Haplotyping

The genotyping data was checked for conformity with Hardy-Wienberg's equilibrium (HWE) as well as for linkage disequilibrium (LD) between the two loci in each study group using the Haploview software (Daly Lab at Broad Institute, USA). LD was described in terms of disequilibrium coefficient (*D*′), correlation coefficient (*r* ^2^), and log of the likelihood odds ratio (LOD), the later being a measure of significance (LOD > 2 is significant). Haplotyping at the subject level was performed using the SimHap software (Centre for Genetic Epidemiology and Biostatistics, University of Western Australia), which employs the estimation-maximization (EM) algorithm for the estimation of haplotypes [12].

### 2.5. Statistical Analysis

Association of allele frequencies, genotypes, or haplotypes with chronic periodontitis was assessed with logistic regression, adjusting for mean plaque index (MPI) and interaction terms if present. When zero frequencies were encountered, exact logistic regression was performed instead. For genotypes and haplotypes, the analysis was performed using the recessive, additive, and dominant models. The odds ratio and 95% confidence intervals were calculated for each model. A  *P*-value ≤0.05 was considered significant. The LogXact software (Cytel Corporation, USA) was used for performing exact logistic regression; SPSS was used for all other analyses.

## 3. Results

### 3.1. The IL-1A C[−889]T Locus Analysis

Table 3 describes the IL-1A C[−889]T allele frequencies, genotype distribution, and HWE status in both study groups. The genotyping data conformed to HWE in the controls as well as the cases (*P* = 0.35 and 1.0, resp.). The T allele was observed at a higher frequency in the cases compared to the controls (55% versus 38.8%); however, the difference was statistically insignificant after adjustment for MPI. Genotype-wise, the T allele showed significant association with sever periodontitis only in the recessive model (TT versus TC + CC genotypes) with an odds ratio (OR) of 6.29.

### 3.2. The IL-1B C[3954]T Locus Analysis

The IL-1B C[3954]T allele frequencies, genotype distribution and HWE status in both study groups are shown in Table 4. There was a significant deviation of genotype distribution from HWE in the controls but not in the cases (*P* = 0.01  and 0.7, resp.). The difference in the T allele frequencies between the cases (47.5%) and the controls (40%) was not statistically significant. Initially, genotype-based analysis did not reveal significant association with periodontitis in any of the models. However, a significant interaction between MPI and the IL-1B C[3954]T genotype was observed and after adjustment for this, the homozygous T allele genotype (recessive model) did show a significant association with the disease (OR = 461, 95% CI: 2.80–7.5E4).

### 3.3. The IL-1 Two-Locus Analysis

The IL-1A C[−889]T and IL-1B C[3954]T loci were found to be in significant LD in the cases (*D*′ = 0.80  and  *r* ^2^ = 0.47) but not in the controls as shown in Table 5. The frequencies of the C-C, C-T, T-C, and T-T haplotypes were 47.5%, 13.8%, 12.5%, and 26.2%, respectively, for the controls, and 41.3%, 3.7%, 11.2%, and 43.8%, respectively, for the cases. Haplotype-based analysis (Table 6) revealed that the C-T haplotype was significantly associated with being a control (protective effect) in the log additive as well as the dominant models (OR = 0.17 and 0.16, resp.). On the other hand, the T-T haplotype in double-dose (recessive model) showed very strong association with chronic periodontitis (OR = 15.6).

## 4. Discussion

The association between the IL-1 gene polymorphisms and chronic periodontitis was first reported in white Caucasians by Kornman et al [9]. Since then, many attempts have been made to reproduce the results in the same as well as other races/ethnic populations [6]. To the best of our knowledge, the current study is the first to involve a Yemeni population and the second to include Arabs, who are usually classified under the Caucasian race [13].

Because of time and financial constraints, it was necessary to keep the sample size to a minimum. However, the study was very carefully designed as to do so without compromising the statistical power. First, stringent diagnostic criteria for cases were used that only subjects with very sever chronic periodontitis were included (5.53 mean CAL), justifying the use of an OR of 3 for sample size calculation. In fact, this case-enrichment approach is recommended for minimizing phenotypic heterogeneity and improving statistical power [14]. Second, controls were matched for age and sex to avoid the need for statistically adjusting for their effects. This is actually an advantage over a number of previous studies in which controls were much younger than cases [15–17]. Finally, smokers and those with diabetes were excluded, eliminating the possibility of interactions and the need for data stratification.

Good quality DNA was obtained from the oral epithelium samples, providing further support for their reliable use as noninvasive alternative to blood samples. In fact, complete concordance in IL-1 genotyping results from oral epithelium and blood samples have been previously demonstrated [18].

The IL-1A T allele frequency in the controls (38.8%) is somewhat similar to that reported in Arabs from Kuwait (35%; [19]) and Jordon (33.7%; [16]) and falls within the range reported in the literature (19–57%) for Caucasians [20–25]. This is in contrast with the very low prevalence (5.6–11.5%) found in subjects of Asian origin [15, 26, 27]. On the other hand, the control IL-1B T allele frequency observed in this study (40%) is considerably higher than the 7.5–29% range reported for Caucasians [20–24]; however, it is close to that reported for Jordanian Arabs (45%) which is the highest in the literature [16]. Again, a much lower prevalence (1.2–4%) has been found in Asians [15, 26, 28]. The IL-1B C[3954]T genotyping data in the controls significantly deviated from HWE; a nonsignificant deviation was also noted for the IL-1A locus. This may be explained, at least in part, by the fact that first-cousin marriage, and therefore non-random mating, is common in Arab societies [29]. However, such a deviation was not observed in previous studies on Arabs [16, 19].

In the current study, the IL-1A C[−889]T and IL-1B C[3954]T polymorphisms showed strong association with sever periodontitis in the TT versus CT + CC genotype contrast (recessive model). This is in line with results from a number of previous reports in which either or both loci showed an association with chronic periodontitis, mostly in its sever form, in the same contrast [24, 30, 31] or in the TT + CT versus CC contrast [25, 32–35]. As with the current study, the association was only observed in nonsmokers. On the other hand, however, several authors have been unable to demonstrate any such association [15, 17, 20, 23, 27, 36].

While this controversy may be correctly attributed to differences in the race/ethnicity of studied populations, especially when comparing Asians with Caucasians, other factors such as differences in study design and data analysis probably account for a greater part of the variation among the reported results. Lopez et al. [22], for example, showed no association between the IL-A C[−889]T polymorphism and chronic periodontitis in a Caucasian Chilean population; however, they were later [30] able to demonstrate a significant association in another, yet, Caucasian Chilean population. The major difference between the two studies was case definition: the latter study included older cases with more sever chronic periodontitis compared to the former (4.2 and 1.9 mm mean CAL, resp.); this substantiates the importance of recruiting extreme phenotypes to enhance statistical power in genetic association studies, especially when small sample size is used. Using a low cutoff for definition of cases may, therefore, explain failure to demonstrate significant associations in some studies [16, 20]. Matching controls for age is another very important aspect of study design that has been missed by a number of authors who failed to show an association between the IL-1 polymorphisms and chronic periodontitis [15–17]. Differences among studies in performing data analysis may be another source of variation. Moreira et al. [33, 34], for example, showed a significant association between the IL-1A C[−889]T and IL-1B C[3954]T polymorphisms, and chronic periodontitis in the TT + TC versus CC contrast (dominant model); however, they did not attempt to do a comparison in the recessive (TT versus TC + CC) or log additive models which would have probably resulted in reporting stronger association as the data suggests. In fact, the current study is the first to explicitly perform comparisons in the three models.

In addition to showing a significant association at the individual locus level, the current study, and for the very first time, shows a strong association between IL-1 polymorphisms and sever chronic periodontitis based on bi-locus haplotype analysis and LD contrast between cases and control; a properiodontitis (T-T) and an antiperiodontitis (C-T) haplotypes were identified, and a significant LD was observed in cases but not in controls. Karasneh et al. [16] and Trevilatto et al. [37] have very recently used a similar approach for data analysis, but they were unable to demonstrate any association, neither did they provide haplotyping or LD data. However, a number of previous studies did report a strong association between the unphased composite genotype (simultaneous carriage of the T allele at the IL-1A C[−889]T and IL-1B C[3954]T loci) and chronic periodontitis [9, 38–40], which is somewhat is in line with the current findings although haplotyping data cannot be directly compared with raw composite genotyping data.

In conclusion, findings from the current study provides further support for an association between the IL-1 polymorphisms and severity of chronic periodontitis, and a preliminary evidence for the usefulness of LD and haplotype-based analysis in exploring for genetic determinants of periodontitis. However, assessing more loci (extended haplotypes) in larger-scale studies is required to explore this further.

## Figures and Tables

**Table 1:** Clinical characteristics of the study groups.

Variable	Cases *n* = 40	Controls *n* = 40
No. of males (%)	21 (52.5%)	20 (50.0%)
Mean age ±SD	43.40 ± 7.06	42.95 ± 5.27
Mean clinical attachment loss ±SD^∗^	05.53 ± 1.15	00.41 ± 0.94
Mean pocket depth ±SD^∗^	03.06 ± 0.60	01.12 ± 0.32
Mean plaque index ±SD^∗^	01.60 ± 0.49	00.97 ± 0.41

^∗^ Difference statistically significant; *t*-test.

**Table 2:** Primer sequences used in the study.

Locus	Primer sequence (5^′^-3^′^)	Reference
IL-1*α*	Forward: TGTTCTACCACCTGAACTAGGC^∗^	[9]
	Reverse: TTACATATGAGCCTTCCATG	
IL-1*β*	Forward: CTCAGGTGTCCTCGAAGAAATCAAA	
	Reverse: GCTTTTTTGCTGTGAGTCCCG	

^∗^ There are additional 5 nucleotides (AAGCT) in the 5^′^ end of the primer according to the reference; since they were found to be noncomplementary to the target and change the amplicon size to 104 bp, they were omitted in this study.

**Table 3:** IL-1A C[−889]T allele frequency, genotype distribution, and HWE status in the cases and controls.

Parameter	Overall *N* = 80	Cases *n* = 40	Controls *n* = 40	OR^¶^ (95% CI)		
				Dominant model	Additive model	Recessive model
C allele	85 (53.1%)	36 (45.0%)	49 (61.2%)		—^a^	
T allele	75 (46.9%)	44 (55.0%)	31 (38.8%)		1.92 (0.86–4.27)^b^	

CC genotype	21 (26.3%)	08 (20.0%)	13 (32.5%)			
TC genotype	43 (53.7%)	20 (50.0%)	23 (57.5%)	1.43 (0.37–5.55)	2.18 (0.89–5.39)	6.29 (1.21–32.6)^∗^
TT genotype	16 (20.0%)	12 (30.0%)	04 (10.0%)			

HWE^c^	+	+	+		N/A	

^¶^OR: odds ratio in the cases compared to the controls; logistic regression adjusting for mean plaque index.

^a^Reference category.

^b^Models are not applicable.

^c^HWE: Hardy-Weinberg equilibrium; (+): consistent with; (−): significant deviation.

^∗^ *P* ≤ 0.05.

**Table 4:** IL-1B C[3954]T allele frequency, genotype distribution, and HWE status in the cases and controls.

Parameter	Overall *N* = 80	Cases *n* = 40	Controls *n* = 40	OR^¶^ (95% CI)		
				Dominant model	Additive model	Recessive model
C allele	90 (56.2%)	42 (52.5%)	48 (60.0%)		—^a^	
T allele	70 (43.8%)	38 (47.5%)	32 (40.0%)		4.20 (0.37–48.0)^b^	

CC genotype	22 (27.5%)	12 (30.0%)	10 (25.0%)			
TC genotype	46 (57.5%)	18 (45.0%)	28 (70.0%)	1.05 (0.02–60.0)	7.38 (0.45–121)	461 (2.80–7.5E4)^∗^
TT genotype	12 (15.0%)	10 (25.0%)	02 (05.0%)			

HWE^c^	+	+	−		N/A	

^¶^OR: odds ratio in the cases compared to the controls; logistic regression adjusting for mean plaque index (MPI) and MPI-genotype interaction.

^a^Reference category.

^b^Models are not applicable.

^c^HWE: Hardy-Weinberg equilibrium; (+): consistent with; (−): significant deviation.

^∗^ *P* ≤ 0.05.

**Table 5:** IL-1A C[−889]T and IL-1B C[3954]T linkage disequilibrium analysis.

Statistic	Overall	Cases *n* = 40	Controls *n* = 40
Disequilibrium coefficient (D^′^)	0.62	0.80	0.44
Correlation coefficient (*r* ^2^)	0.34	0.47	0.18
Log of likelihood odds ratio (LOD)	>2	>2	<2

^∗^Statistically significant when >2.

**Table 6:** IL-1 two-locus haplotype analysis.

Haplotype	Bi-allelic	Cases *n* = 40	Controls *n* = 40	OR^¶^ (95% CI)		
				Dominant model	Additive model	Recessive model
	O/O	14 (35.0%)	08 (20.0%)			
C-C	C-C/O	19 (47.5%)	26 (65.0%)	0.31 (0.08–1.17)	0.86 (0.36–2.09)	4.03 (0.77–21.2)
	C-C/C-C	07 (17.5%)	06 (15.0%)			

	O/O	37 (92.5%)	30 (75.0%)			
C-T	C-T/O	03 (07.5%)	09 (22.5%)	0.16 (0.03–0.85)^∗^	0.17 (0.03–0.83)^∗^	0.72 (0.00–28.0)
	C-T/C-T	00 (00.0%)	01 (02.5%)			

	O/O	32 (80.0%)	30 (75.0%)			
T-C	T-C/O	07 (17.5%)	10 (25.0%)	0.92 (0.24–3.50)	0.93 (0.25–3.50)	0.04 (0.001–INF)
	T-C/T-C	01 (02.5%)	00 (00.0%)			

	O/O	13 (32.5%)	19 (47.5%)			
T-T	T-T/O	19 (47.5%)	21 (52.5%)	1.73 (0.54–5.56)	2.55 (0.95–6.87)	15.6 (1.58–INF)^∗^
	T-T/T-T	08 (20.0%)	00 (00.0%)			

O: other haplotype.

^¶^OR: odds ratio in the cases compared to the controls; logistic regression adjusting for mean plaque index.

^∗^ *P* ≤ 0.05.
